# Reply: ‘Pre-treatment levels of circulating free IGF-1 identify NSCLC patients who derive clinical benefit from figitumumab’

**DOI:** 10.1038/bjc.2011.413

**Published:** 2011-10-11

**Authors:** A Gualberto, M L Hixon, M Pollak

**Affiliations:** 1 Department of Pathology and Laboratory Medicine, Warren Alpert Medical School at Brown University, Providence, RI 02903, USA; 2 Department of Medical Oncology, McGill University, Montreal, Quebec H3T 1E2, Canada

**Sir**,

We read with great interest the Letter to the Editor from Shimokawa *et al* (2011) that reported significant associations between the expressions of the insulin-like growth factor type I receptor (IGF-IR) and those of E-cadherin and *γ*-catenin in non-small cell lung cancer (NSCLC) biopsies (Shimokawa *et al*, 2011). These data reproduce previous observations from our group. We have also observed a correlation between the expressions of IGF-IR and E-cadherin, particularly in NSCLC tumours with a high degree of differentiation (Gualberto *et al*, 2010). Furthermore, using unsupervised Bayesian clustering of epithelial-to-mesenchymal transition (EMT)- and IGF-IR-related markers, we identified three NSCLC subsets that resembled the steps of the EMT and we named epithelial-like, transitional-like and mesenchymal-like (Gualberto *et al*, 2010). Several markers of the IGF-IR pathway such as nuclear insulin receptor substrate-1 were overexpressed in the transitional subset and a higher objective response rate to the combination of chemotherapy and the anti-IGF-IR antibody figitumumab was observed in patients with transitional tumours (Gualberto *et al*, 2010). Thus, we agree with Shimokawa *et al* (2011) that analysis of tumour EMT status may contribute to a better understanding of the sensitivity to anti-IGF-IR therapy.

Shimokawa *et al* (2011) also pointed out that we have reported an inverse correlation in NSCLC patients between serum IGF-1 and tumour E-cadherin levels (Gualberto *et al*, 2011). We speculate that the fact that tumour E-cadherin is directly correlated with tumour IGF-IR and inversely correlated with serum IGF-1 may indicate differential functions for the non-activated (low or no ligand) *vs* the activated (in the presence of IGFs) IGF-1 receptor. Evidence supports the involvement of the IGF-IR in both differentiation and cellular growth and metastasis, with engagement of the receptor by IGFs-inducing neo-expression of mesenchymal markers, E-cadherin downregulation and cell migration (reviewed in Julien-Grille *et al*, 2005). These observations may have therapeutic implications. Of note, our key finding that high serum (free) IGF-1 levels may identify a subset of NSCLC patients who preferentially benefit from anti-IGF-IR therapy has now been independently corroborated by other groups (Goto *et al*, 2011; Ramalingam *et al*, 2011).
